# The distribution of Coronal Plane Alignment of the Knee (CPAK) phenotypes in the Malaysian population and their correlation with demographic variables

**DOI:** 10.1186/s42836-024-00281-z

**Published:** 2024-12-04

**Authors:** Wai Kit Wong, Azliana Abu Bakar Sajak, Hwa Sen Chua

**Affiliations:** 1 Department of Orthopaedics and Traumatology, Hospital Ampang, Ampang, Selangor 68000 Malaysia; 2 Clinical Research Centre, Sunway Medical Centre, Bandar Sunway, Subang Jaya, Selangor 47500 Malaysia; 3 Orthopaedic Centre of Excellence, Sunway Medical Centre, Bandar Sunway, Subang Jaya, Selangor 47500 Malaysia

**Keywords:** CPAK, Functional alignment, Constitutional alignment, Individualized alignment strategy

## Abstract

**Background:**

Only 80% of patients are satisfied with their outcomes post-TKA. Mounting attention has been paid to constitutional limb alignment and individualized alignment strategies in recent years. MacDessi et al. proposed the CPAK classification, which takes into account the patients’ arithmetic hip-knee-ankle axis (aHKA) and joint line obliquity (JLO). In this study, we aimed to establish local demographic data, compare them with published data, and assess their correlations with modifiable variables.

**Methods:**

A total of 500 end-stage osteoarthritic knees subjected to TKA were radiologically analyzed. The lateral distal femoral angle (LDFA) and medial proximal tibial angle (MPTA) were calculated from long limb radiographs before the aHKA and JLO were derived and a CPAK phenotype was assigned. Demographic data were harvested and analyzed for possible correlations.

**Results:**

There were 160 males (32%) and 340 females (68%), with a mean age of 66.42 years (range, 47–88). The mean MPTA was 85.8° (± 3.0)°, and the mean LDFA was 87.6° (± 2.4)°. The average aHKA was a varus of 1.8° (± 4.2)°, and the average JLO was 173.4° (± 3.45)°. The most common CPAK phenotype was Type 1 (43.4%). The Intraclass Correlation Coefficient demonstrated excellent reliability (> 0.9). No correlation existed between CPAK phenotypes and age, height, weight, or body mass index (BMI), but CPAK phenotype was significantly correlated with gender.

**Conclusion:**

An urban Malaysian population with osteoarthritic knees was found to be constitutionally varus, with the most common phenotype being varus aHKA with an apex-distal JLO. Constitutional alignment is not influenced by factors such as age, height, weight, or BMI.

**Level of Evidence:**

Retrospective Observational Study-III.

## Introduction

Various alignment strategies are available when performing total knee arthroplasty (TKA) for end-stage knee osteoarthritis (OA) [1, 2]. The decision regarding which alignment principles to adhere to is largely dependent on the beliefs and preferences of the surgeon, as there is still no conclusive evidence of the superiority of one alignment strategy over the others [2, 3]. John Insall first described the principles of Mechanical Alignment (MA) in 1985, whereby the femoral and tibial cuts are made perpendicular to the mechanical axis, resulting in a neutral lower limb axis and a neutral joint line [4]. Insall believed that these principles result in an even distribution of joint stresses, leading to the best balance of function restoration and implant longevity [4]. Early biomechanical research by Hollister on knee kinematics paved the way for the eventual development of the concept of Kinematic Alignment (KA), which was further perpetuated by the introduction of navigation systems [5, 6]. KA principles aim to match the implants to recreate the pre-arthritic joint orientation of the patient. As component alignment would theoretically better match the bony anatomy and native soft tissue envelope, soft tissue releases are reduced, thus potentially improving knee balance [3, 6]. More recently, the development of robotic systems has introduced the concept of Functional Alignment (FA), whereby permissible lower limb alignment has been expanded from conventional neutral ± 3° to accommodate the attainment of optimal implant positioning and equal gap balance [7, 8].

Only 80% of patients are satisfied after TKA despite the fact that it is the gold standard treatment [9, 10]. With so much research ongoing, in hopes of improving functional outcomes and patient satisfaction, there should be an improved system for reporting data that is encompassing yet easily applicable. MacDessi et al. recently proposed a new classification system based on long limb radiographs, i.e., the Coronal Plane Alignment of the Knee (CPAK) classification [11]. This classification considers the arithmetic Hip-Knee-Ankle axis (aHKA) and joint line obliquity (JLO), thus providing a better estimate of the constitutional limb alignment of patients. The published literature has provided demographic data for osteoarthritic knees in Australia [11], India [12], Japan [13], Turkey [14], South Africa [15], and France [16]. There are no known data regarding the CPAK classification and distribution of coronal alignment in the Malaysian and Southeast Asian populations.

In this study, we aimed to establish the CPAK distribution of osteoarthritic knees in an urban Malaysian population and compare it with data from other populations already published in the literature and to determine whether there are any correlations between the CPAK classification and age, as well as modifiable variables such as height, weight, and BMI.

## Materials and methods

### Patients

**Fig. 1:**
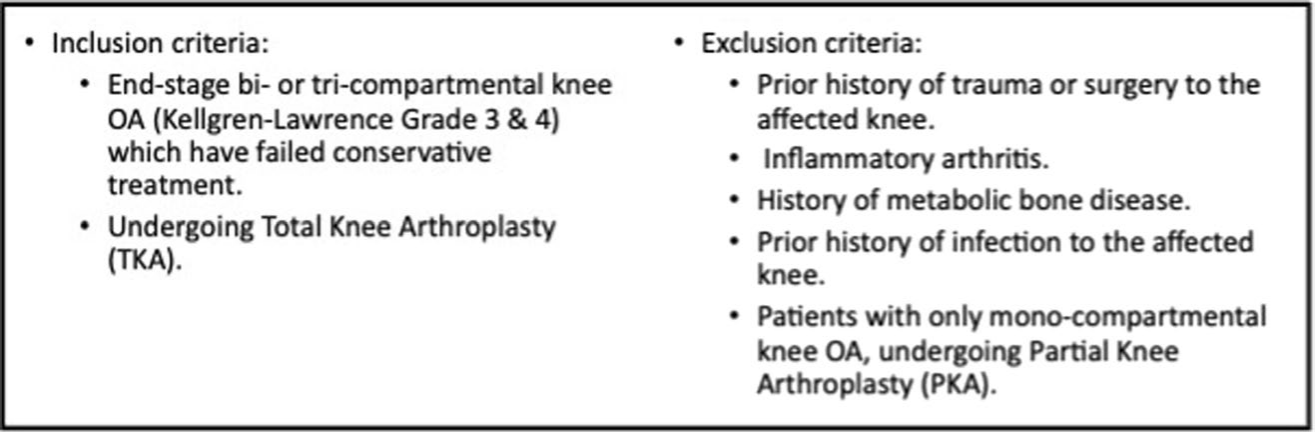
Inclusion and exclusion criteria for patient enrollment in this study

Upon obtaining ethical clearance from the Sunway Medical Centre Independent Research Ethics Committee (SREC No. 018/2023/IND/ER), we began our retrospective review of all patients who underwent a TKA from October 2021 until February 2024. We included the first 500 knees that fulfilled the inclusion and exclusion criteria listed in Fig. 1 and had complete demographic data.

### Radiological assessment

All patients routinely received long limb radiographs as part of preoperative planning at our center. The radiographs were obtained in accordance with the established protocol outlined by Paley [17]. Radiographic images are stored on a PACS system, and all measurements were performed via built-in measurement tools.

The mechanical lateral distal femoral angle (LDFA) was defined as the lateral angle subtended by the mechanical axis of the femur and the joint line of the distal femur. The mechanical medial proximal tibial angle (MPTA) is defined as the medial angle between the mechanical axis of the tibia and the joint line of the proximal tibia [11].

The mechanical axis of the femur is defined by a line connecting the center of the femoral head and center of the knee, and the mechanical axis of the tibia is represented by a line between the center of the knee and center of the ankle. The center of the femoral head is identified by using the concentric circle method, and the center of the ankle is the midpoint of the talus [12]. As per the reference study by MacDessi et al., the constitutional alignment of the patient is approximated by the “arithmetic HKA, aHKA”, which is derived from the following formula: aHKA = MPT.A − LDFA [18]. A negative value denotes varus alignment, and a positive value denotes valgus. Neutral alignment is taken at 0° ± 2°, inclusive [11].

Joint line obliquity (JLO), the second key parameter in the determination of the CPAK classification, is calculated according to the formula JLO = MPTA + LDFA [11]. The degree of obliquity was measured in relation to the floor with both legs of the patient planted in a double-leg stance. The JLO was taken to be neutral or parallel to the horizontal if JLO = 180° ± 3°, inclusive. A value of ≤ 176.9° was deemed an apex-proximal joint line, whereas a JLO of ≥ 183.1° is apex-distal [11].

**Fig. 2:**
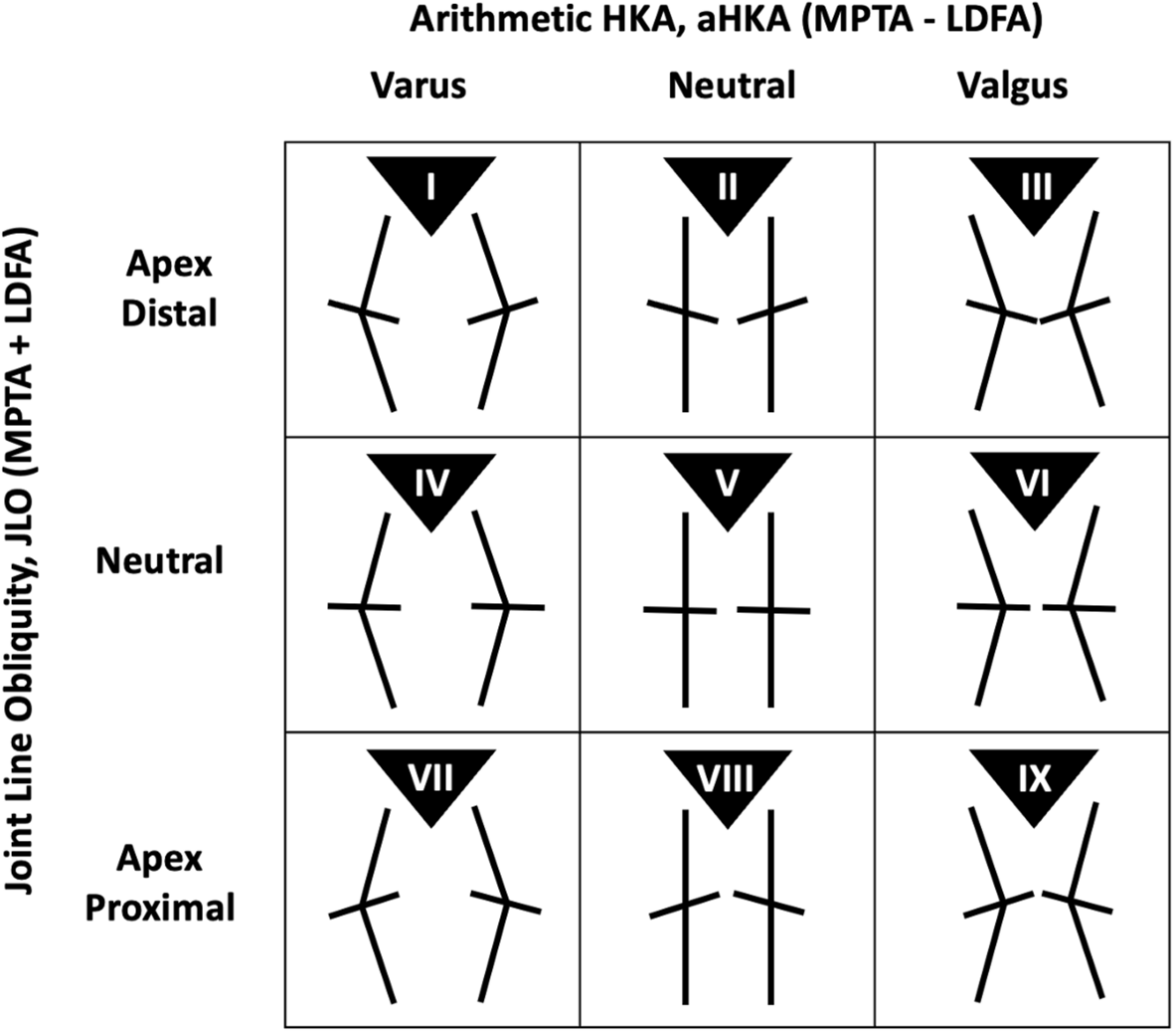
Coronal Plane Alignment of the Knee (CPAK) classification with 9 distinct phenotypes

Once the aHKA and JLO were measured, the patients were then assigned to 1 of the 9 possible CPAK classification groups, as depicted in Fig. 2.

### Data analysis

The Intraclass Correlation Coefficient was utilized to assess the intra-observer and inter-observer reproducibility of these measurements on a randomly generated subgroup of 30 knees. The measurements were performed separately by a senior surgeon (CHS), who is a fellowship-certified arthroplasty surgeon; a junior surgeon (WWK), who commences fellowship training soon, and a trainee (KS). The measurements were then repeated at one-week intervals for another two sets of readings.

The data were then analyzed by employing SPSS version 29 (International Business Machine Corporation; Armonk, New York, NY, USA), with significance set at *P* < 0.05. Using the central limit theorem for a large sample (> 30) with equal variance as a basis, the Kruskal–Wallis test was conducted for comparison, and chi-square tests were used to assess the associations between the variables [19, 20].

## Results

Recruited in our study were 160 males (32%) and 340 females (68%), with a mean age of 66.42 years (range, 47–88). There were 254 right (50.8%) and 246 left (49.2%) knees. The average BMI was 27.6 kg/m^2^, with most patients (46.6%) being overweight.

The reliability of intra-observer and inter-observer measurements was assessed in terms of the Intraclass Correlation Coefficient, which demonstrated excellent reliability (score > 0.9), as illustrated in Table 1.

**Table 1:** Assessment of intra-observer and inter-observer reliability of measurements

Parameter	Rater	Intraclass Correlation Coefficient							
		**Intra-observer**	**95% Confidence Interval**		***P***	**Inter-observer**	**95% Confidence Interval**		***P***
			**Lower Bound**	**Upper Bound**			**Lower Bound**	**Upper Bound**	
LDFA	1	0.984	0.972	0.992	< 0.001	0.991	0.982	0.995	< 0.001
	2	0.993	0.986	0.996	< 0.001				
	3	0.984	0.970	0.992	< 0.001				
MPTA	1	0.980	0.963	0.990	< 0.001	0.984	0.970	0.992	< 0.001
	2	0.986	0.973	0.993	< 0.001				
	3	0.978	0.959	0.989	< 0.001				

Two-way mixed effects model was used where people effects were random, and measures effects were fixed (absolute agreement definition used)

*LDFA* lateral distal femoral angle, *MPTA* medial proximal tibial angle

**Fig. 3:**
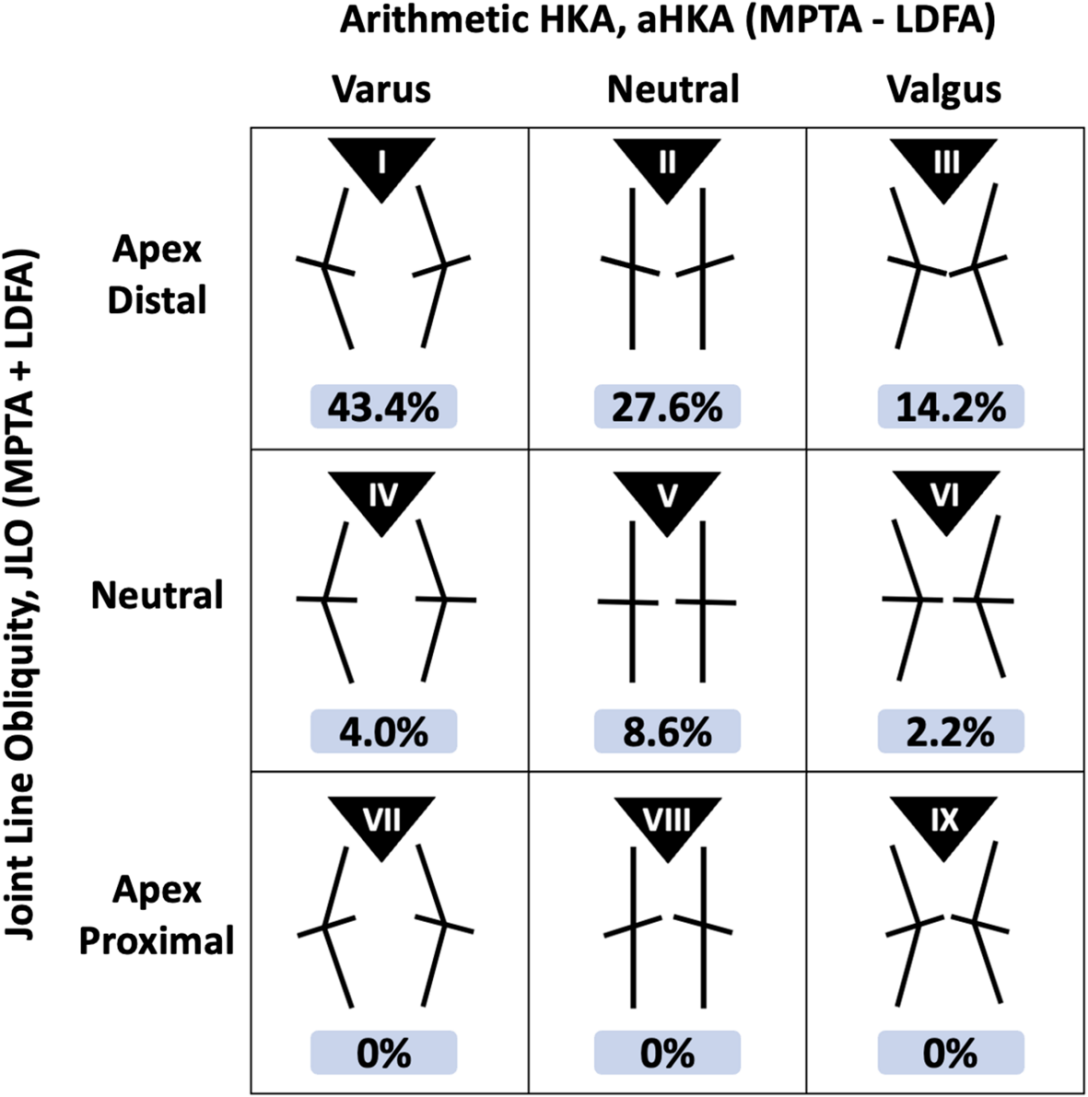
Distribution of the study population according to the CPAK classification

**Fig. 4:**
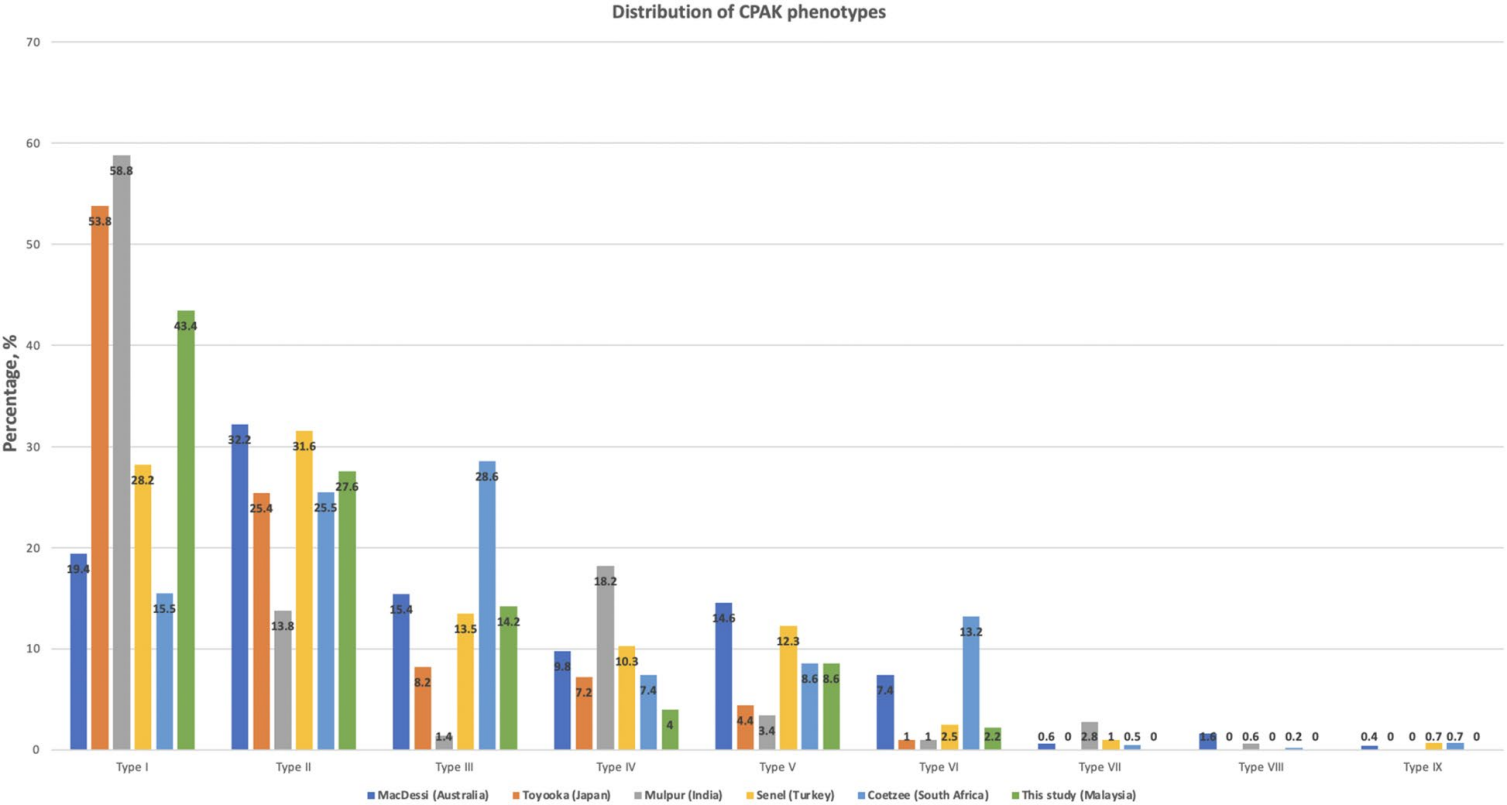
Comparison of the CPAK phenotype distributions among different geographical populations that have been reported thus far

The mean MPTA was 85.8° (± 3.0)°, and the mean LDFA was 87.6° (± 2.4)°. The average aHKA was a varus of 1.8° (± 4.2)°, and the average JLO was 173.4° (± 3.45)°. The most common CPAK phenotype was Type 1 (43.4%). No patients had Types 7, 8, or 9 phenotypes. Figure 3 shows the complete distribution of our study population, and Fig. 4 illustrates the differences in the CPAK distributions among the various geographical populations that have been published in the literature.

Table 2 compares the CPAK distribution along with demographic data for patients with arthritic knees from different geographical regions in literature so far.

**Table 2:** The distribution of CPAK phenotypes and radiographic measurements of arthritic patients from various geographical regions

	**MacDessi (Australia)** ***n* = 500**	**Mulpur (India)** ***n* = 500**	**Toyooka (Japan)** ***n* = 500**	**Sappey-Marinier (France)** ***n* = 1078**	**Senel (Turkey)** ***n* = 408**	**Coetzee (South Africa)** ***n* = 608**	**This study (Malaysia)** ***n* = 500**
Gender							
Male	190 (38%)	152 (30%)	95 (19%)	780 (72%)	141 (48%)	76 (22%)	160 (32%)
Female	310 (62%)	348 (70%)	405 (81%)	298 (28%)	155 (52%)	268 (78%)	340 (68%)
Age^a^, years	66 (Range, 44–88)	62.3 ± 8.2	75.1 ± 8.0	71.3 ± 8.0	54.5 ± 7.9	68.4 ± 9.2	66.4 ± 6.7
aHKA^a^, degrees	− 0.8 ± 2.8	− 6.85 ± 5.0	− 3.5 ± 4.8	− 1.7 ± 3.5	− 1.4 ± 3.9	1.0 ± 4.8	1.8 ± 4.2
JLO^a^, degrees	NA	173.5 ± 5.0	172.4 ± 3.8	176.0 ± 4.5	174.6 ± 3.7	175.2 ± 3.4	173.4 ± 3.5
mLDFA^a^, degrees	88.1 ± 2.1	90.2 ± 3.6	88.0 ± 2.9	88.9 ± 3.0	88.0 ± 2.9	87.2 ± 3.0	87.6 ± 2.4
mMPTA^a^, degrees	87.3 ± 2.1	83.3 ± 3.4	84.4 ± 3.3	87.1 ± 2.8	86.6 ± 2.6	88.2 ± 2.8	85.8 ± 3.0
CPAK distribution, %							
Type I	19.4	58.8	53.8	33.4	28.2	15.5	43.4
Type II	32.2	13.8	25.4	19.5	31.6	25.5	27.6
Type III	15.4	1.4	8.2	10.6	13.5	28.6	14.2
Type IV	9.8	18.2	7.2	10.2	10.3	7.4	4.0
Type V	14.6	3.4	4.4	18.9	12.3	8.6	8.6
Type VI	7.4	1.0	1.0	6.3	2.5	13.2	2.2
Type VII	0.6	2.8	0	0.4	1.0	0.5	0
Type VIII	1.6	0.6	0	0.6	0	0.2	0
Type IX	0.4	0	0	0.1	0.7	0.7	0

*aHKA* arithmetic Hip-Knee-Ankle axis, *JLO* Joint line obliquity, *mLDFA* Mechanical lateral distal femoral angle, *mMPTA* mechanical medial proximal tibial angle, *CPAK* Coronal Plane Alignment of the Knee classification, *NA* Not available

^a^Presented as mean ± SD

No correlation was found between CPAK phenotypes and age, height, weight, or BMI, but there was a statistically significant correlation between the CPAK phenotype and sex (Table 3).

**Table 3:** Correlation between CPAK phenotype and variables

Variables	H score	Degree of freedom, df	*P*
Age^α^	1.988	5	0.851
Height^α^	0.745	5	0.980
Weight^α^	4.347	5	0.501
BMI^α^	4.689	5	0.455
	**X**^**2**^** score, *N* = 500**		
Gender ^β^	12.242	5	0.027

^α^Kruskal-Wallis test; *P* < 0.05: statistically significant at 95% CI

^β^Chi-Square test, *P* < 0.05: statistically significant at 95% CI

## Discussion

Our study analyzed the phenotypical distribution of osteoarthritic knees in a Malaysian urban community based on the Coronal Plane Alignment of the Knee (CPAK) classification [11]. There was a tendency for the population to have a varus constitutional alignment, with 47.4% of the population being classified as varus. The most common phenotype was Type I, which represents a varus aHKA and an apex-distal JLO. No correlation existed between CPAK phenotypes and age, height, weight, and BMI.

MacDessi et al. first proposed the CPAK classification based on two cohorts of patients, with the arthritic group being patients from Australia and the normal group being recruited from Belgium [11]. The healthy arm consisted of 250 healthy subjects having both knees evaluated, thus amounting to the 500 knees they based their non-arthritic population on. The arthritic arm consisted of 500 consecutive Australian patients who underwent total knee arthroplasty. Hsu et al. reported CPAK data on 214 healthy knees of a Taiwanese population; Toyooka et al. published data on Japanese patients with arthritis; Mulpur et al. furnished data on Indian patients; Sappey-Marinier wrote on French patients; Şenel et al. published a study on Turkish patients and Coetzee provided South African data [12–16, 21]. To our knowledge, our study was the first to report the distribution of CPAK phenotypes in Malaysian and Southeast Asian patients and to evaluate its possible correlation with age, height, weight, and BMI.

The phenotype with the highest prevalence in our study was Type I (43.4%), which was consistent with the Type 1 prevalence rates of 53.8% and 58.8%, respectively, in the Japanese and Indian cohorts of patients [13]. In a study by MacDessi et al., Type II (neutral aHKA with an apex-distal JLO) was the most common phenotype among both the healthy (39.2%) and arthritic (32.2%) populations. This observation suggests that the Asian population has a more pronounced constitutional varus as opposed to their Australian and European counterparts. Prior to the introduction of the CPAK classification, Song et al. reported in 2015 that there was an increased incidence of constitutional varus among Korean women compared with the Western population [22]. Our findings corroborated the findings of Mulpur and Toyooka, further strengthening this observation. It is also interesting to note the predominance of the type 3 phenotype (28.6%) within the South African population and that 41.8% of their cohort had a valgus aHKA [15]. These findings highlight the significant variability of constitutional alignment among various geographical regions and that aiming for a limb alignment of neutral ± 3° as per mechanical alignment principles may not result in a consistently favorable outcome.

MacDessi and colleagues also evaluated the intercompartmental pressure difference by using a wireless pressure sensor to measure the difference in pressure between the medial and lateral compartments at various flexion angles [11]. They also assessed the number of bone recuts required to achieve a balanced knee between knees operated using the alignment principles of Kinematic Alignment (KA) vs. Mechanical Alignment (MA). For Type I knees, restoration of the joint line by adopting the principles of KA allowed for better balance at all flexion angles compared to those in the MA group. With type IV knees, the KA arm had better balance at extension and mid-flexion. The KA arm also required fewer bone cuts to achieve a balanced knee [11]. These findings suggest that the restoration of constitutional varus is an important consideration that may improve functional outcomes for patients, thus raising patient satisfaction rates post-TKA, since only approximately 80% of patients are satisfied presently [9, 10]. With the increasing utilization of robotic systems in TKA, which offer precise control and precision in terms of implant placement in the coronal, sagittal, and axial planes as well as real-time feedback on gap balance based on minute intraoperative changes, the recognition of the prevalence of constitutional varus and, subsequently, the aim of its restoration should be closely considered to improve post-TKA patient satisfaction [23–25]. The senior author (HS Chua) has since evolved his practice to increase the adoption of the principles of Functional Alignment whereby the femoral and tibial coronal angles are independently allowed a range of neutral ± 3°, but when taken together, the permitted HKA axis is limited to neutral ± 5° [7, 8]. This expansion of the permissible lower limb axis facilitates the attainment of optimal implant positioning in all 3 planes as well as an equal gap balance while simultaneously respecting the inherent constitutional alignment that patients have.

Only 8.6% of the patients in the study population showed a Type V phenotype. Other studies yielded varying prevalence rates: MacDessi’s Australian cohort reported a rate of 14.6%, Toyooka's Japanese cohort 4.4%, the Indian population 3.4%, French patients 18.9%, Turkish patients 12.3%, and South Africans 8.6% [11–16]. The adoption of mechanical alignment principles aims to achieve neutral alignment with a neutral joint line but only restores constitutional alignment for a small subset of patients. While this approach has resulted in excellent survivorship, this departure from what they have become accustomed to may be a contributing factor to the current dissatisfaction rates.

The results indicated that the constitutional alignment remains unaffected by non-modifiable variables such as age, alongside modifiable factors such as height, weight, and BMI. By leveraging robotic assistance, surgeons achieve precise control, enabling greater deviation from neutral alignment while ensuring that the patient's constitutional alignment is respected and that the peri-articular soft tissue envelope is preserved. These measures are anticipated to enhance patients’ experience with a knee that feels more natural, potentially enhancing postoperative outcomes and satisfaction levels.

Based on the 95% confidence interval of the Intraclass Correlation Coefficient (ICC) estimate, values less than 0.5, between 0.5 and 0.75, between 0.75 and 0.9, and greater than 0.90 are indicative of poor, moderate, good, and excellent reliability, respectively. Our excellent intra-observer and inter-observer scores mirrored those of MacDessi, Mulpur, Şenel, and Coetzee, lending further strength to the utilization of the CPAK classification as a simple, reliable, and reproducible system in communication and research pertaining to lower limb alignment [11, 12, 14, 15].

This study is not without limitations. Although we followed a strict protocol while obtaining standing long limb radiographs, we were unable to fully exclude rotational errors. Despite this, the high interclass and intraclass reliability validated the methodology we undertook. An alternative is to utilize a long-limb CT scan to replace the radiographs. However, in patients who do not wish to undergo a robotic TKA using the CT-based MAKO system, the additional radiation from a CT scan is not justifiable. Furthermore, a CT scan is obtained with the patient supine, thus removing the effects of weight-bearing on the actual severity of their deformities. Finally, although this study established a baseline distribution according to the CPAK classification and the prevalence of constitutional varus in our population, further research is needed to correlate phenotypes and postoperative outcomes.

## Conclusion

An urban Malaysian population with osteoarthritic knees was found to be constitutionally varus, with the most common phenotype being varus aHKA with an apex-distal JLO. Additionally, constitutional alignment was not influenced by factors such as age, height, weight, and BMI. Respecting the constitutional alignment of patients undergoing TKA may improve postoperative outcomes and satisfaction rates.

## Data Availability

The datasets used and/or analyzed during the current study are available from the corresponding author on reasonable request.
